# The Beta-Glucan Receptor Dectin-1 Recognizes Specific Morphologies of *Aspergillus fumigatus*

**DOI:** 10.1371/journal.ppat.0010042

**Published:** 2005-12-09

**Authors:** Chad Steele, Rekha R Rapaka, Allison Metz, Shannon M Pop, David L Williams, Siamon Gordon, Jay K Kolls, Gordon D Brown

**Affiliations:** 1 Department of Pediatrics, Children's Hospital of Pittsburgh, University of Pittsburgh School of Medicine, Pittsburgh, Pennsylvania, United States of America; 2 Department of Surgery, James H. Quillen College of Medicine, East Tennessee State University, Johnson City, Tennessee, United States of America; 3 Sir William Dunn School of Pathology, University of Oxford, Oxford, United Kingdom; 4 Institute of Infectious Disease and Molecular Medicine, University of Cape Town, Cape Town, South Africa; University of California at Los Angeles, United States of America

## Abstract

Alveolar macrophages represent a first-line innate host defense mechanism for clearing inhaled *Aspergillus fumigatus* from the lungs, yet contradictory data exist as to which alveolar macrophage recognition receptor is critical for innate immunity to *A. fumigatus*. Acknowledging that the *A. fumigatus* cell wall contains a high beta-1,3–glucan content, we questioned whether the beta-glucan receptor dectin-1 played a role in this recognition process. Monoclonal antibody, soluble receptor, and competitive carbohydrate blockage indicated that the alveolar macrophage inflammatory response, specifically the production of tumor necrosis factor-α (TNF-α), interleukin-1α (IL-1α), IL-1β, IL-6, CXCL2/macrophage inflammatory protein-2 (MIP-2), CCL3/macrophage inflammatory protein-1α (MIP-1α), granulocyte-colony stimulating factor (G-CSF), and granulocyte monocyte–CSF (GM-CSF), to live *A. fumigatus* was dependent on recognition via the beta-glucan receptor dectin-1. The inflammatory response was triggered at the highest level by *A. fumigatus* swollen conidia and early germlings and correlated to the levels of surface-exposed beta glucans, indicating that dectin-1 preferentially recognizes specific morphological forms of *A. fumigatus*. Intratracheal administration of *A. fumigatus* conidia to mice in the presence of a soluble dectin-Fc fusion protein reduced both lung proinflammatory cytokine/chemokine levels and cellular recruitment while modestly increasing the *A. fumigatus* fungal burden, illustrating the importance of beta-glucan–initiated dectin-1 signaling in defense against this pathogen. Collectively, these data show that dectin-1 is centrally required for the generation of alveolar macrophage proinflammatory responses to *A. fumigatus* and to our knowledge provides the first in vivo evidence for the role of dectin-1 in fungal innate defense.

## Synopsis

Individuals with defective immune systems are highly susceptible to infection by parasites, bacteria, viruses, and fungi. Infection by the opportunistic fungal organism *Aspergillus fumigatus* can be particularly severe in this population. Because many pathogenic microorganisms, including *A. fumigatus,* enter the body through the lung, it is important to understand the function of its immune system. The alveolar macrophage is one of the first cell types to come in contact with inhaled pathogens. An intense area of research is how lung immune cells—i.e., alveolar macrophages—recognize inhaled pathogens and respond to them. Steele et al. recently discovered that alveolar macrophages express a receptor on their surface, dectin-1, that is essential in recognizing and responding to inhaled fungal pathogens. They now have investigated the interaction between dectin-1 and *A. fumigatus* to determine how the dectin-1 receptor orchestrates the alveolar macrophage response. They found that alveolar macrophages respond poorly to *A. fumigatus* when the dectin-1 receptor is blocked. Also, in animal experiments, blocking dectin-1 renders the animals more susceptible to infection with *A. fumigatus*. This study may lay the foundation for developing new and novel strategies to combat infections caused by *A. fumigatus*.

## Introduction

Individuals with compromised immune systems are at high risk for acquired invasive fungal infections. *Aspergillus fumigatus,* the etiological agent of invasive pulmonary aspergillosis (IPA), is a ubiquitous mold that causes severe, invasive, life-threatening disease in patients who are severely immunocompromised. Disease acquisition includes such risk factors as neutropenia and impaired neutrophil function and myeloablative-immunosuppressive therapies associated with hematopoietic stem-cell transplantation [1]. Despite available anti-fungal therapy, the prognosis of IPA remains poor, and mortality ranges from 30% to 90% [2,3]. This is thought to be due in part to the relatively small arsenal of effective anti-fungal drugs, some of which cause severe nephrotoxicity—specifically, amphotericin B, which is associated with response rates of between 10% and 40% [4]. IPA has risen dramatically over the past several decades due to the consistent increase in immunosuppressed patients, and by the early 1990s 60% of invasive fungal infections diagnosed at autopsy were IPA [5]. It must also be stated that IPA is not only associated with stem-cell transplantation, but also presents in whole-organ transplantation, primarily lung and heart, with mortality rates of 68% to 78% [6]. *A. fumigatus* is also the etiological agent of allergic bronchopulmonary aspergillosis, an allergic airway disease characterized by persistent bronchial inflammation and bronchiectasis [7].

Upon inhalation of *A. fumigatus* conidia from the environment, alveolar macrophages rapidly ingest and attempt to clear the invading pathogen. Conidia that escape the fungicidal activities of alveolar macrophages begin to germinate, leading to the rapid recruitment of neutrophils, which subsequently promote anti-hyphal defenses [8,9]. A major focus in innate immunity and host-pathogen interactions in the past decade has been elucidation of the receptors involved in the recognition and response to pathogens, the most characterized of which are the toll-like receptors (TLRs). However, in the context of macrophage–*A. fumigatus* interactions, there is no clear role for the TLRs in recognition and responsiveness. TLR2 and TLR4 are the most studied. However, the data on TLR2 are conflicting in that several reports have shown roles for and against its importance in host defense against this pathogen. For example, macrophages from TLR2^−/−^ mice produce less tumor necrosis factor-α (TNF-α) [10] and CXCL2/MIP-2 [11] in response to *A. fumigatus,* whereas antibody-mediated blockage of TLR2 had no effect on TNF-α production [12] and TLR2^−/−^ mice challenged with *A. fumigatus* survived better than wild-type control mice and had higher lung levels of TNF-α [13]. A role for TLR4 in the inflammatory response to *A. fumigatus* conidia, but not hyphae, has also been demonstrated [14,15], suggesting that TLR4 is critical for recognition of different *A. fumigatus* morphologies. TLR4^−/−^ mice challenged with *A. fumigatus* have increased susceptibility compared with control mice [13], although this is not associated with defects in TNF-α production, as it was unaffected by TLR4^−/−^ deficiency [13]. Other studies show that TLR4 signaling is essential for the anti-fungal effector activity of neutrophils [16], but not Kupffer cells [17], against both conidia and hyphae of *A. fumigatus*. In addition, many of these studies investigating the role of TLRs and *A. fumigatus* recognition have not been performed with alveolar macrophages, and thus it is uncertain if the mechanisms described are representative of these cells, which have a unique phenotype.

Non-TLRs are also important in innate recognition of *A. fumigatus*. The cell wall of *A. fumigatus* is known to contain galactomannan moieties that are thought to be covalently linked to the non-reducing ends of beta-1,3–glucan side chains [18]. Accordingly, several studies have described mannose- or mannan-specific receptors in the uptake of *A. fumigatus* conidia by phagocytic cells [19]. Studies have identified the C-type lectin DC-SIGN (dendritic-cell-specific, ICAM-3-grabbing nonintegrin) as being involved in the binding of *A. fumigatus* conidia to human macrophages and dendritic cells [20].

The *A. fumigatus* cell wall is also rich in beta-1,3–glucan moieties, and although receptors for these carbohydrates, including CR3, have been implicated, the role of these receptors in innate immune response to this organism is unclear [21]. We have previously shown that recognition of cell-wall beta glucan plays an important role in the induction of inflammatory mediators by macrophage populations in response to *Pneumocystis carinii* and *Candida albicans* [22,23]. Dectin-1 is a 43-kDa, type II transmembrane receptor containing a single cytoplasmic immunoreceptor tyrosine activation motif and is the predominant macrophage receptor for beta-1,3 glucans [23–25]. As dectin-1 is highly expressed on resident alveolar macrophages, we examined the role of this receptor in response to *A. fumigatus,* and show here that dectin-1 is centrally involved in generating inflammatory responses to specific morphological forms of this organism both in vitro and in vivo.

## Results/Discussion

### Dectin-1 Is Involved in the Macrophage Cytokine and Chemokine Response to *A. fumigatus*

**Figure 1:**
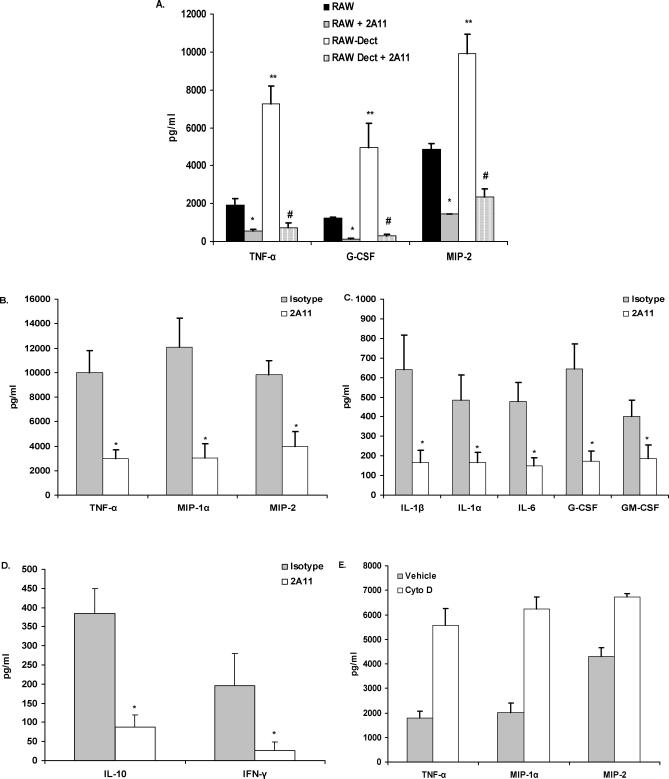
Dectin-1 Dependent Cytokine and Chemokine Production in Response to *A. fumigatus* (A) Native RAW 264.7 macrophages or RAW 264.7 over-expressing murine dectin-1 were pretreated with isotype or 2A11 and co-cultured for 24 h with live *A. fumigatus.* Supernatant cytokine and chemokine levels were determined by Bio-Plex or ELISA. (A) illustrates cumulative results from five separate experiments. Asterisks, double asterisks, and the hash sign represent significant differences between RAW and RAW + 2A11, RAW and RAW-Dect, and RAW-Dect and RAW-Dect + 2A11, respectively (*p* < 0.05). Data are expressed as mean pg/ml + SEM. (B–D) Identical experimental design with alveolar macrophages. (B–D) illustrate cumulative results from five separate experiments. Asterisks represent significant differences between isotype and 2A11 (*p* < 0.05). Data are expressed as mean pg/ml + SEM. (E) Alveolar macrophages were pre-treated with 10-μM cytochalasin D and co-cultured with live *A. fumigatus* for 16 h. Supernatant cytokine and chemokine levels were determined by Bio-Plex or ELISA. (E) illustrates cumulative results from three separate experiments. Data are expressed as mean pg/ml + SEM.

Extensive reports have shown that zymosan, a beta-glucan–rich, yeast-derived particle, and the beta-glucan–containing fungal organisms *C. albicans* and *P. carinii* [22,25] bind to the dectin-1 beta-glucan receptor leading to phagocytosis and proinflammatory cytokine production [25,26]. *A. fumigatus* similarly possesses a cell wall significantly made up of beta glucans [18]; thus, we questioned whether macrophage interactions with *A. fumigatus* involved dectin-1. The results in Figure 1A show that RAW 264.7 cells, a macrophage cell line that was established from a tumor induced by Abelson murine leukemia virus [27], can produce a number of cytokines and chemokines in response to live *A. fumigatus* after 24 h of co-culture, and that this response is greatly enhanced in RAW 264.7 macrophages transduced to over-express dectin-1. In both cell types, inhibition of dectin-1 function with the monoclonal antibody 2A11 [26] significantly blocked these responses. Control experiments stimulated RAW 264.7 cells with the TLR ligands LPS and Pam(3)Cys in the presence or absence of 2A11. Results showed that dectin-1–blockage did not impair the TNF-α and MIP-2 response to these stimulants (unpublished data). Thus, dectin-1 can recognize and respond to live *A. fumigatus*.

Innate immune cells of the lung, particularly alveolar macrophages, are critical for recognizing and reacting to *A. fumigatus* [8,9]. Since we have previously shown that dectin-1 is expressed at high levels on alveolar macrophages [22,28], we assessed their response to live *A. fumigatus*. The results in Figure 1B and 1C show that co-culture of live *A. fumigatus* with alveolar macrophages for 24 h led to production of TNF-α, CCL3/MIP-1α, CXCL2/MIP-2, IL-1β, IL-1α, IL-6, G-CSF, and GM-CSF, all of which were significantly attenuated by blocking dectin-1 with the monoclonal antibody 2A11 [26]. We observed little spontaneous production of cytokines and chemokines by unstimulated alveolar macrophages (e.g., spontaneous production of TNF-α, CCL3/MIP-1α, and CXCL2/MIP-2 was 54.7 ± 20, 186 ± 45, and 122 ± 25 pg/ml, respectively). T helper type-1 cell-mediated immunity is essential for optimal pulmonary host defense against fungal infections [29]. Innate cells, such as alveolar macrophages, play a central role in aiding the development of T helper type-1 responses [29]. In our studies, we found that alveolar macrophages stimulated with *A. fumigatus* had dectin-1–dependent induction of IFN-γ (Figure 1D), suggesting that dectin-1–ligation by *A. fumigatus* may also promote the generation of T helper type-1 immunity. IL-12 was also induced by *A. fumigatus,* but not found to be dectin-1–dependent (233 ± 83 pg/ml and 71 ± 22 pg/ml for isotype and 2A11, respectively, *p =* 0.0902). We also observed dectin-1–dependent induction of IL-10, a critical cytokine for regulating the pulmonary inflammatory response [30].

Previous studies indicated that cytochalasin D–treated macrophages stimulated with the fungal particle zymosan had enhanced TNF-α production, indicating that internalization was not required for dectin-1–mediated cytokine and chemokine production in RAW macrophages [23]. Although zymosan is employed as a representative fungal particle, it is not clear whether its use predicts the subsequent events associated with dectin-1 ligation by a live, intact fungal organism such as *A. fumigatus.* Experimental studies have shown that unstimulated alveolar macrophages are quite efficient at internalizing *A. fumigatus* conidia, a process that requires actin polymerization [31,32]. We questioned whether blocking actin polymerization would affect the ability of alveolar macrophages to produce inflammatory mediators in response to *A. fumigatus*. We found that alveolar macrophages pretreated with cytochalasin D retained the inflammatory response to live *A. fumigatus* (Figure 1E), and that the production of TNF-α, MIP-1α, and MIP-2 was exacerbated. Fluorescent deconvolution microscopy performed to assess the internalization of *A. fumigatus* in vehicle versus cytochalasin D-treated alveolar macrophages indicated efficient uptake of fluorescein isothiocyanate–conjugated conidia in vehicle-treated, but not cytochalasin D–treated, alveolar macrophages [20,31,32] (Figure S1). We did not observe non-specific induction of cytokines/chemokines by unstimulated alveolar macrophages in the presence of cytochalasin D. Moreover, lactate dehydrogenase (LDH) analysis of co-culture supernatants indicated that cytochalasin D concentration employed in these studies was not cytotoxic to alveolar macrophages (unpublished data). The heightened response in cytochalasin D-treated cultures is likely to be a result of prolonged stimulation at the cell surface, as observed previously with zymosan [33]. These results therefore suggest that alveolar macrophage cytokine and chemokine production in response to live *A. fumigatus* is mediated by dectin-1 and does not require organism uptake.

### Role of TLR2 in Dectin-1–Dependent Alveolar Macrophage Responses to *A. fumigatus*

Dectin-1–mediated inflammatory responses to the fungal particle zymosan have been shown to be dependent on TLR2 [23,34]. However, the role of TLR2 in the inflammatory response to *A. fumigatus* is less clear, with reports both supporting and arguing against its role in the inflammatory response [10–13]. To address the role of dectin-1 in TLR2-mediated responses to *A. fumigatus,* we co-cultured alveolar macrophages isolated from wild-type C57BL/6 and TLR2^−/−^ mice with *A. fumigatus* for 24 h, followed by analysis of cytokine and chemokine levels in co-culture supernatants. Data presented in Table 1 indicate that only TNF-α production was significantly affected (*p* < 0.05) by TLR2 deficiency in 24-h alveolar macrophage–*A. fumigatus* co-cultures. However, reductions were observed for all cytokines examined. Although reduced, TLR2^−/−^ alveolar macrophages could still produce nanogram concentrations of TNF-α in response to this fungal pathogen, suggesting that TLR2 was not absolutely required for the production of TNF-α, but did appear to be required for optimal dectin-1–mediated TNF-α production. Inhibition of dectin-1, which is expressed equally on these cells (unpublished data), resulted in more than 80% reduction in most cytokines (the exception being IL-1α and GM-CSF) including TNF-α, in both C57BL/6 and TLR2^−/−^ alveolar macrophage–*A. fumigatus* co-cultures. We have also made similar observations with the dectin-1–dependent fungal organisms *P. carinii* (unpublished data). Although previous studies with zymosan have suggested an accessory role of TLR2 in dectin-1–mediated responses [23,34], acknowledging our *A. fumigatus* and *P. carinii* data, we hypothesize that zymosan may contain a ligand with high specificity for TLR2, one that is present at lower amounts in the cell wall of *A. fumigatus* and *P. carinii*. Our data support this hypothesis, since all cytokines examined with TLR2^–/–^ alveolar macrophages were moderately, though not always significantly, reduced in response to these two organisms. Together, these results suggest that TLR2 plays an accessory role with dectin-1 in mediating the alveolar macrophage inflammatory response to live *A. fumigatus*.

**Table 1:**
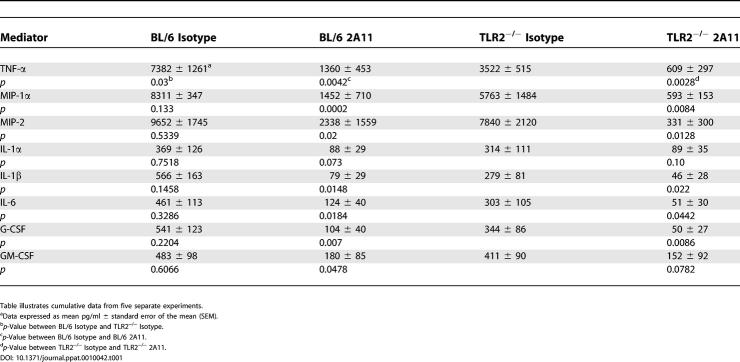
Cytokine and Chemokine Responses of Naïve Alveolar Macrophages from TLR2-Deficient Mice to *A. fumigatus*

### Macrophage Cytokine and Chemokine Production Is Dependent on Recognition of Exposed Beta Glucan in *A. fumigatus* Swollen Conidia and Early Germlings

**Figure 2:**
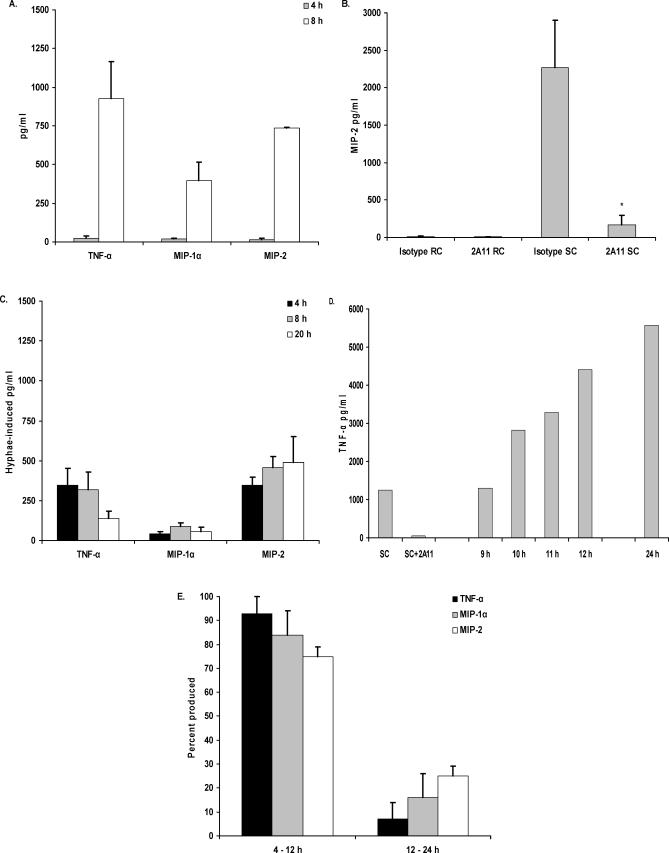
Cytokine and Chemokine Production Is Dependent on Beta-Glucan Recognition in *A. fumigatus* SC and Pre-Competent Hyphae Alveolar macrophages were added to (A) live *A. fumigatus* RC and incubated for 4 h and 8 h, (B) live RC or SC and incubated for 4 h in the presence of isotype or 2A11, or (C) live *A. fumigatus* hyphae and incubated for 4 h, 8 h, and 24 h. (D and E) Alveolar macrophages were added to *A. fumigatus* RC and incubated for 24 h. Samples for cytokine analysis were taken between 9 h and 12 h, and at 24 h, as indicated. Also shown in (D) is a representative 4-h response to pre-generated SC, and inhibition of this response by 2A11. Thereafter, cytokine and chemokine levels were determined in supernatants by Bio-Plex or ELISA. Percentage data expressed in (E) were calculated by dividing the pg/ml cytokine/chemokine level in 12-h samples by that in 24-h samples. Asterisks represent significant differences between isotype (rat IgG) and 2A11 (*p* < 0.05). (A–C,E, and F) are cumulative results from four experiments.

Disease caused by *A. fumigatus* is initiated when conidia, termed “resting conidia” (RC), are inhaled into the lung. Upon entering the lung, RC go through phenotypic changes that lead to swelling, now termed “swollen conidia” (SC), followed by germination [35]. As alveolar macrophages are thought to be the most critical in early stages after inhalation [8,9], the alveolar macrophage inflammatory response to various morphological stages of *A. fumigatus* was investigated. Examining the kinetics of cytokine production after exposure to live *A. fumigatus* RC, we observed that while TNF-α, CCL3/MIP-1α, or CXCL2/MIP-2 were readily detectable after 8 h of co-culture, very low concentrations (<30 pg/ml) of these mediators were detected at 4 h (Figure 2A). Furthermore, we did not observe an effect of dectin-1 blockage in cytokine levels at 4 h; however, the levels at 8 h were significantly blunted (e.g., TNF-α levels at 4 h and 8 h were reduced from 16 pg/ml and 373 pg/ml to 7 pg/ml and 2 pg/ml in the presence of 2A11, respectively). As this suggested that the *A. fumigatus* RC did not have beta glucans exposed within the first 4 h of culture, we examined the response to live *A. fumigatus* SC or hyphae, using TNF-α, CCL3/MIP-1α, or CXCL2/MIP-2 as an indicator of dectin-1–mediated recognition. In contrast to *A. fumigatus* RC, a 4-h incubation of alveolar macrophages with *A. fumigatus* SC (RC incubated for 6 h at 37 °C prior to the addition of macrophages) efficiently induced CXCL2/MIP-2 production, which could be abrogated by blocking dectin-1 (Figure 2B). It must be stated that these co-cultures were initiated with the *A. fumigatus* SC morphology, but this morphology did convert to early germlings over the course of the 4-h incubation period. Alveolar macrophages added to live *A. fumigatus* hyphae (RC allowed to germinate for 24 h prior to the addition of macrophages), however, induced only low levels of TNF-α, CCL3/MIP-1α, and CXCL2/MIP-2, even after prolonged stimulation (Figure 2C). Although previous studies have shown higher cytokine responses to *A. fumigatus* hyphae using peritoneal macrophages and non-viable hyphae [10–12,14,15], our studies are nevertheless in agreement with live, mature hyphae inducing TNF-α production. We do not think that the lower amounts of cytokines induced by live, mature hyphae in our study was a result of macrophage death, as we did not observe increases in LDH or caspase-3 levels (measured in cell lysates) compared to unstimulated supernatants (unpublished data). Possible explanations for the lower levels of cytokine induction in our studies may be differences in macrophage-to-hyphae ratios, our usage of live versus heat-killed/fixed hyphae, or the tissue source of the macrophages (peritoneal versus alveolar). As we had observed significantly higher cytokine levels at 24 h of co-culture (see Figure 1B) than at 8 h of co-culture (Figure 2A), we performed a time-course analysis of cytokine levels produced by alveolar macrophages in response to *A. fumigatus*. We observed that more than 75% of the TNF-α, CCL3/MIP-1α, and CXCL2/MIP-2 concentrations observed at 24 h were elicited between 9 h and 12 h (Figure 2D and 2E), during conidial swelling and germination. These data suggest that less than 25% of the total cytokine/chemokine response in a 24-h culture is produced after 12 h, further suggesting that dectin-1 recognizes beta-glucan moieties of *A. fumigatus* morphologies that are present well before 12 h.

**Figure 3:**
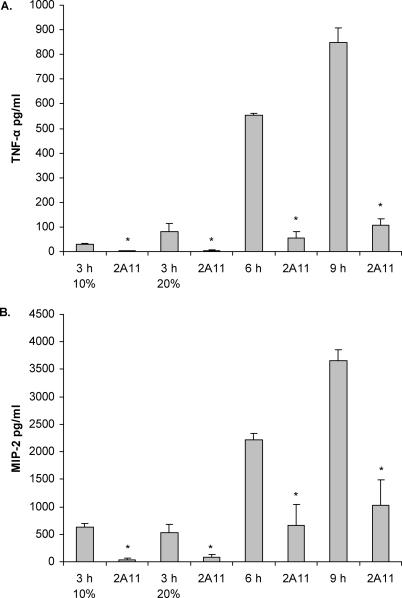
Cytokine and Chemokine Induction in Response to Different Heat-Killed Morphologies of *A. fumigatus* *A. fumigatus* was grown for 3 h (in 10% or 20% serum), 6 h, or 9 h and thereafter subjected to heat-killing. Alveolar macrophages were added and incubated in the presence or absence of isotype or 2A11 for 6 h. Thereafter, cytokine and chemokine levels were determined in supernatants by Bio-Plex or ELISA. Asterisks represent significant differences between isotype (rat IgG) and 2A11 (*p* < 0.05). Figure 3 shows cumulative results from three experiments. Data are expressed as mean pg/ml + SEM.

To further determine *A. fumigatus* morphological induction of the inflammatory response mediated by dectin-1, we cultured *A. fumigatus* for 3 h, 6 h, and 9 h, subjected them to heat-inactivation, and thereafter added the heat-killed conidia to alveolar macrophages for 6 h. Results presented in Figure 3 show that 6-h, heat-killed organisms elicited TNF-α (Figure 3A) and CXCL2/MIP-2 (Figure 3B) from alveolar macrophages in a dectin-1–dependent manner. Additional studies employing 6-h, ethanol-killed organisms showed similar dectin-1–dependent induction (for TNF-α, 1,208 pg/ml versus 65 pg/ml in 2A11-treated cells; for CXCL2/MIP-2, 1,817 pg/ml versus 722 pg/ml for 2A11-treated cells). Moreover, 9-h, heat-killed *A. fumigatus,* or early germlings, were also efficient in eliciting TNF-α and CXCL2/MIP-2 from alveolar macrophages in a dectin-1–dependent manner. Previous reports have also suggested that *A. fumigatus* conidia cultured in 20% serum swell within 3 h [31]. We therefore examined the ability of *A. fumigatus* conidia cultured for 3 h in 10% and 20% serum followed by heat-inactivation to induce dectin-1–dependent TNF-α and CXCL2/MIP-2 production. Although 3-h, heat-killed *A. fumigatus* originally cultured in 20% serum elicited more TNF-α than the 10% serum *A. fumigatus* and in a dectin-1–dependent manner, this induction was less than 15% of that induced by either 6-h, heat-killed organisms or 9-h, heat-killed early germlings (Figure 3A). There was no difference in the level of CXCL2/MIP-2 induction between the 3 h in 10% and 20% serum morphologies. Taken collectively, these results indicate that alveolar macrophages recognize beta glucans via dectin-1 when *A. fumigatus* conidia begin to swell and vigorously respond to SC and early germlings.

**Figure 4:**
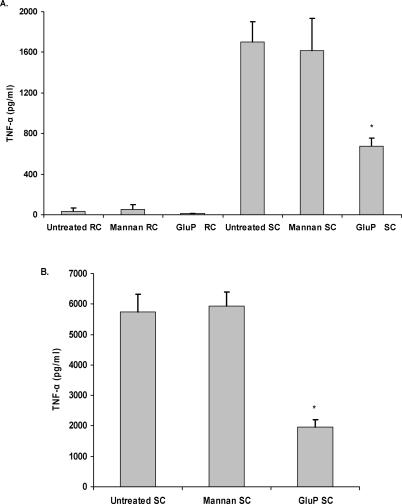
Competitive Carbohydrate Blockage Indicates No Role of Mannan Receptors in the Inflammatory Response Alveolar macrophages (A) or RAW 264.7 macrophages over-expressing dectin-1 (B) were pretreated with 250 μg/ml *S. cerevisiae*-derived mannan or 100 μg/ml glucan phosphate and then added to RC or SC. Thereafter, cytokine and chemokine levels were determined in supernatants by Bio-Plex or ELISA. Each graph illustrates cumulative results from four separate experiments. Asterisks represent significant differences between untreated and glucan-phosphate treated (*p* < 0.05).

The cell wall of *A. fumigatus* is known to contain galactomannan moieties [36], and several studies have implicated mannose- or mannan-specific receptors, including DC-SIGN (dendritic-cell-specific, ICAM-3-grabbing nonintegrin) and the long pentraxin PTX3, in the recognition of *A. fumigatus* [19,20]. To address the possible role of a mannose- or mannan-specific receptor in the induction of inflammatory response by SC, we pretreated alveolar macrophages with *Saccharomyces cerevisiae*–derived mannan [20] prior to their addition to SC and observed no effect, whereas addition of a soluble beta glucan, glucan phosphate [23,26] significantly inhibited TNF-α production (Figure 4A).

To confirm these findings, we examined the response of RAW 264.7 macrophages over-expressing dectin-1 to *A. fumigatus* SC. Similar to what we had observed in the alveolar macrophages, there was little to no production of TNF-α in response to RC, whereas SC resulted in significant induction of TNF-α (Figure 4B). The production of TNF-α could be significantly inhibited by pretreatment with 2A11 or the dectin-1 antagonist glucan phosphate, but not by mannan. Taken collectively, these results indicate that cytokine and chemokine production by alveolar macrophages in response to *A. fumigatus* is mediated by dectin-1, but that this occurs only during the swelling and germination of conidia.

### Specific Binding of Dectin-1 to *A. fumigatus* SC

**Figure 5:**
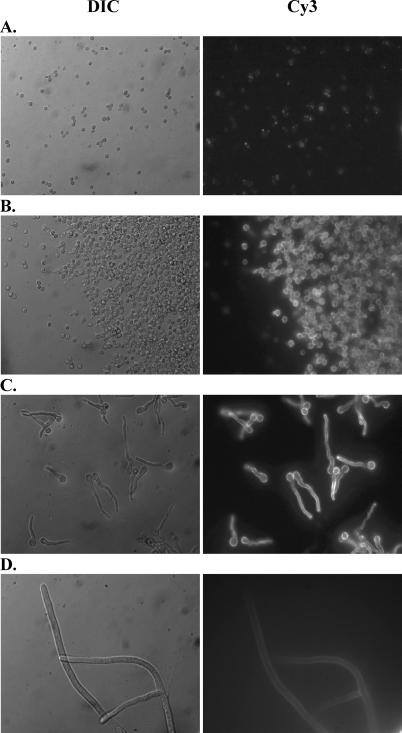
Heightened Binding of Dectin-1 to *A. fumigatus* SC A soluble fusion protein consisting of the extracellular carbohydrate recognition domain of dectin-1 fused with the Fc portion of murine IgG1 (s-dectin-mFc) was constructed and incubated with live *A. fumigatus* RC and SC. Binding of s-dectin-mFc was detected by Cy3-conjugated, goat anti-mouse IgG antibody followed by imaging with a Zeiss Axioplan 2 upright fluorescent deconvolution microscope (Zeiss), and images were captured using 3i Slidebook Version 4.0 software (Optical Analysis). Representative micrographs show s-dectin-mFc binding to *A. fumigatus* grown for 2 h (A), 6 h (B), 10 h (C), and 24 h (D). Left lane images are differential interference contrast (DIC) images, and right lane images are Cy3 staining. Magnification is 630 × oil emersion for all frames.

To further verify the interaction between dectin-1 and beta-glucan moieties of *A. fumigatus,* we constructed a soluble fusion protein consisting of the extracellular portion of dectin-1 containing the carbohydrate recognition domain [25] fused with the Fc portion of murine IgG1 (termed s-dectin-mFc). Using s-dectin-mFc as a probe to examine the levels of exposed beta glucan on the surface of the different morphologies, we observed by fluorescent deconvolution microscopy that RC (2 h) showed very low beta-glucan staining with s-dectin-mFc (Figure 5A), whereas SC (6 h) (Figure 5B) and early germlings (10 h) (Figure 5C) stained brightly. Beta-glucan moieties exposed on mature hyphae (24 h) (Figure 5D) were present and recognized by s-dectin-mFc, but at a much lower intensity than SC and early germlings. The beta-glucan staining pattern of SC and early germlings–pre-competent hyphae appeared more uniform with beta-glucan moieties exposed over the surface of the conidia and along the early hyphal extension. In contrast, RC beta-glucan staining was qualitatively less and more irregular and punctuated. Beta-glucan staining on live, mature hyphae appeared qualitatively lower than early germlings, but was present nonetheless. The staining pattern of beta-glucan exposure on live, mature hyphae that we observed is in general agreement with a report showing beta-glucan staining with the monoclonal 2G8 [37]. Thus, these data support the concept that beta-glucan moieties are recognized by the carbohydrate recognition domain of dectin-1 initially in *A. fumigatus* SC and continually recognized through the conversion to early germlings. After live, mature hyphae have completely formed, the dectin-1 ligands are still present and recognized by dectin-1, but seemingly at lower levels and therefore do not stimulate as vigorous a response. The amount of beta-glucan exposure of these four morphological forms correlated to the level of cytokine induced by RC (see Figures 2A and 3), SC/early germlings (see Figures 2B and 3), and live, mature hyphae (see Figure 2C).

### In Vivo Administration of a s-Dectin-1-Fc Fusion Protein Abrogates Inflammation in Response to *A. fumigatus*

**Figure 6:**
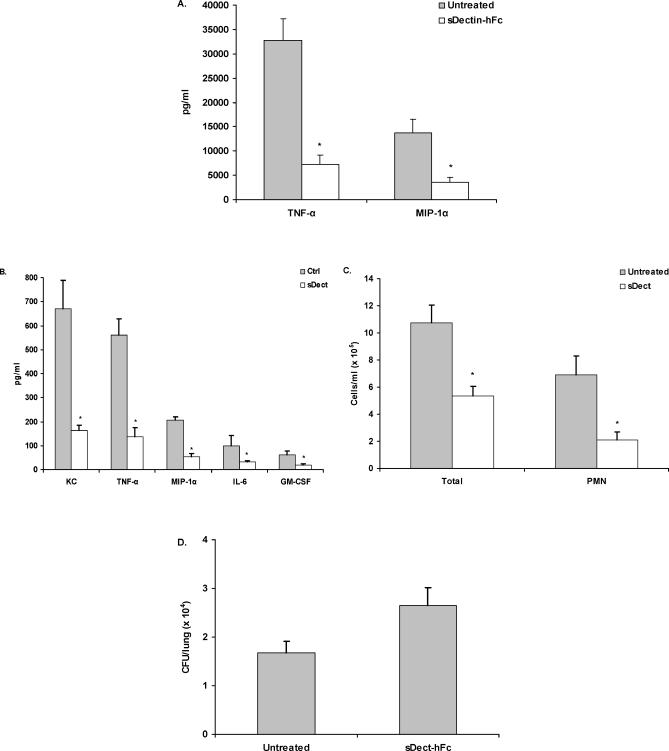
Blockage of Dectin-1-Mediated Inflammation In Vivo after *A. fumigatus* Lung Challenge C57BL/6 mice were intratracheally administered live *A. fumigatus* conidia in the presence or absence of s-dectin-hFc. Mice were sacrificed 24 h later, and a BAL was performed. (A) Alveolar macrophages were co-cultured for 24 h with live *A. fumigatus* in the presence or absence of s-dectin-hFc (10 μg/ml)*.* Supernatant cytokine and chemokine levels were determined by Bio-Plex or ELISA. (A) illustrates cumulative results from four separate experiments. Asterisks represent significant differences between untreated and s-dectin-1–containing wells (*p* < 0.05). Data are expressed as mean pg/ml + SEM. (B) Cytokine and chemokine levels in clarified BAL fluid from untreated and soluble (s-dect)–treated mice was measured by Bio-Plex. (B) illustrates representative results from three independent experiments (*n* = 5–7 mice per group). Asterisks represent significant differences between untreated and s-dectin-1–treated mice (*p* < 0.05). Data are expressed as mean pg/ml + SEM. (C) Total cell counts (Total) in BALF fluid as enumerated on a hemacytometer. Neutrophil (PMN) concentrations were determined by calculating the percentages of neutrophils in three to five sets of 100 cells and multiplying the percentage with the total BALF cell number. (D) Lungs were excised from non-lavaged–untreated and s-dectin-hFc–treated mice, homogenized, followed by serial 1:10 dilutions, and plated onto potato dextrose agar. CFU/lung were determined after incubating the plates for 24 h at 37 °C. (D) illustrates representative results from three independent experiments (*n* = 5–7 mice per group).

To examine the role of dectin-1 in vivo, we constructed a second s-dectin-1 fusion protein, containing mutated human IgG1 Fc (s-dectin-hFc) [38]**.** Preliminary studies confirmed that s-dectin-hFc could abrogate TNF-α and CCL3/MIP-1α production by alveolar macrophages in vitro (Figure 6A). *A. fumigatus* conidia were intratracheally administered to mice in the presence or absence of s-dectin-hFc, and a bronchoalveolar lavage (BAL) was performed after 24 h. By 24 h, detectable levels of CXCL1/KC, TNF-α, CCL3/MIP-1α, IL-6, and GM-CSF (Figure 6B) were found in BAL fluid. In contrast, mice that received *A. fumigatus* in the presence of s-dectin-hFc had significantly lower concentrations of each cytokine and chemokine (*p* < 0.05). Control experiments examining the specificity of s-dectin-hFc indicated that mice administered LPS in the presence or absence of s-dectin-hFc for 24 h had no differences in BAL-fluid TNF-α levels (unpublished data). As CXCL1/KC and CCL3/MIP-1α have been shown to be essential for the recruitment of innate immune cells such as neutrophils during IPA [39,40], we hypothesized that their reduction may lead to a difference in cellular recruitment to the infected lungs. Indeed, mice that had received *A. fumigatus* in the presence of s-dectin-hFc had fewer total recruited cells in the BAL fluid as determined by differential counting as well as fewer absolute neutrophil numbers when compared with mice challenged with *A. fumigatus* in the absence of s-dectin-hFc (Figure 6C). Quantitative plate count analysis indicated that mice receiving s-dectin-hFc at the time of inoculation had modestly higher (approximately 35%) *A. fumigatus* organisms levels 24 h after challenge (Figure 6D; *p* = 0.0502 by two-tailed *t* test, *p =* 0.0281 by the two-tailed Mann-Whitney test). Thus, these data indicate that administration of a s-dectin-1 fusion protein in vivo inhibits the inflammatory response to *A. fumigatus,* leading to reductions in innate cell recruitment to the lung and subsequent impairment of lung clearance of *A. fumigatus.*

*A. fumigatus–*beta-glucan moieties induce inflammation, both in vitro and in vivo, which is to our knowledge the first such report. We identified the receptor on alveolar macrophages responsible for the inflammatory response as the beta-glucan receptor dectin-1. Our studies show that ligation of dectin-1 on alveolar macrophages to unmasked beta-glucan moieties of live *A. fumigatus* swollen and early germlings leads to the potent induction of a variety of proinflammatory cytokines and chemokines, which is involved in the recruitment of neutrophils. These results provide critical insight into one of the earliest recognition events after inhalation of *A. fumigatus* and show the importance of alveolar macrophage–associated, beta-glucan–initiated, dectin-1 signaling in generating the appropriate inflammatory signals in response to *A. fumigatus*.

## Materials and Methods

### Mice.

Male C57BL/6 mice, 6–8 wk of age, were purchased from National Cancer Institute, National Institutes of Health (Bethesda, Maryland, United States). Toll-like-receptor-2–deficient (TLR2^−/−^) mice were generated on the 129SvJ × C57BL/6 background and were backcrossed to the C57BL/6 strain as previously described [41]. All animals were housed in a specific pathogen-free facility and handled according to institutionally recommended guidelines.

### Preparation of *A. fumigatus* conidia.

*A. fumigatus* isolate 13073 (ATCC, Manassas, Virginia, United States) was maintained on potato dextrose agar for 5–7 d at 37 °C. Conidia were harvested by washing the culture flask with 50 ml of sterile, phosphate-buffered saline supplemented with 0.1% Tween 20. The conidia were then passed through sterile gauze followed by passage through a sterile 40 μm nylon membrane to remove hyphal fragments, and then enumerated on a hemacytometer.

### Macrophage populations.

RAW 264.7 macrophages over-expressing dectin-1 were generated and maintained as previously described [23]. For alveolar macrophage isolation, male C57BL/6 or TLR2^−/−^ mice were anesthetized with intraperitoneal pentobarbital and sacrificed by exsanguination. Thereafter, lungs were lavaged through an intratracheal catheter with pre-warmed (37 °C) calcium- and magnesium-free PBS supplemented with 0.6 mM EDTA. A total of 10 ml was used in each mouse in 0.5-ml increments with a 30-s dwell-time. The lavage fluids were pooled and centrifuged at 300 × g for 10 min, and the cells collected for *A. fumigatus* co-culture. To ensure that each cell preparation was enriched for macrophages, 2.5 × 10^4^ cells were cytospun onto slides and stained with hematoxylin and eosin. Cell preparations were >98% enriched for alveolar macrophages.

### *A. fumigatus*-macrophage co-culture for cytokine and chemokine induction.

Alveolar macrophages or RAW-dectin macrophages [23] (3 × 10^5^) were pre-treated with the anti–dectin-1 antibody 2A11 (5 μg/ml) or isotype for 30 min [26] and thereafter co-cultured with *A. fumigatus* conidia at a ratio of 1:1 for various times in a 96-well plate at 37 °C, 5% CO_2_. Controls included alveolar or RAW macrophages cultured in medium alone. Thereafter, the contents of each well were collected and the supernatants analyzed for IL-1α, IL-1β, IL-2, IL-3, IL-4, IL-5, IL-6, IL-10, IL-12p40, IL-12p70, IL-17, IFN-γ, GM-CSF, G-CSF, TNF-α, MIP-1α, RANTES, and KC levels using the Bio-Plex Protein Array System (Bio-Rad, Hercules, California, United States) as per manufacturer's instructions. MIP-2 concentrations were determined using a commercially available ELISA kit (R&D Systems, Minneapolis, Minnesota, United States) as per manufacturer's instructions. To determine the response to SC, *A. fumigatus* RC were cultured for 6 h at 37 °C, 5% CO_2_ and allowed to swell [10,31,42]. Thereafter, macrophages were added for an additional 4 h. Controls for these studies included alveolar macrophages added to RC for 4 h. To determine the response to *A. fumigatus* hyphae, *A. fumigatus* conidia were cultured for 24 h at 37 °C, 5% CO_2_ prior to the addition of macrophages for 4 h, 8 h, or 24 h. Spontaneous cytokine and chemokine production in unstimulated cultures was subtracted from stimulated cultures in order to calculate the net concentration induced by *A. fumigatus.* To determine whether macrophage cell death occurred in co-cultures, supernatants were analyzed for LDH levels using an LDH kit (Sigma, St. Louis, Missouri, United States) as per manufacturer's instructions. Caspase 3 levels in cell lysates were also analyzed using the EnzChek Caspase 3 Assay Kit containing the rhodamine 110 bis-(N-CBZ-L-aspartyl-Lglutamyl-L-valyl-L-aspartic acid amide) (Z-DEVD–R110) substrate (Molecular Probes, Eugene, Oregon, United States) as per manufacturer's instructions. In specific experiments, macrophages were pretreated with cytochalasin D (10 μM), 250 μg/ml mannan (both from Sigma, St. Louis, Missouri, United States), or 100 μg/ml glucan phosphate [23,26] for 30 min at room temperature prior to addition to RC or SC. For analysis of responses to heat-killed *A. fumigatus, A. fumigatus* conidia were cultured for 3 h, 6 h, or 9 h at 37 °C, 5% CO_2_ followed by heat-killing at 100 °C for 10 min [10]. Thereafter, heat-killed *A. fumigatus* were co-cultured with alveolar macrophages at a ratio of 1:1 for 6 h at 37 °C, 5% CO_2_ in a 96-well plate. Some experiments employed *A. fumigatus* organisms killed by 70% ethanol treatment for 30 min at room temperature. All killed *A. fumigatus* were plated onto potato dextrose agar at 37 °C for 48 h to confirm negative growth. In specific experiments, RAW 264.7 macrophages were cultured with LPS (100 ng/ml) or Pam(3)Cys (10 μg/ml) (both from InvivoGen, San Diego, California, United States) in the presence or absence of 2A11 for 16 h, followed by analysis of cytokine and chemokine levels in supernatants.

### Analysis of *A. fumigatus* internalization.

Alveolar macrophages were isolated as described above and adhered to poly-L-lysine-coated glass slides (Polysciences Inc., Warrington, Pennsylvania, United States) for 60 min at 37 °C. After being washed, separated slides were incubated with dimethyl sulfoxide (DMSO, vehicle) or cytochalasin D (10 μM) for 60 min at 37 °C, followed by incubation for 60 min with fluorescein isothiocyanate-labeled *A. fumigatus* conidia (0.1 mg/ml FITC for 60 min at room temperature) [20]. After being washed, slides were counterstained with 4,6-diamidino-2-phenylindole,dihydrochloride (DAPI, Molecular Probes, Eugene, Oregon, United States) nucleic-acid stain (0.4 μg/ml, 10 min at room temperature), followed by application of Prolong (Molecular Probes) mounting media. Slides were analyzed on a Zeiss Axioplan 2 upright fluorescent deconvolution microscope (Carl Zeiss, Oberkochen, Germany), and images were captured using 3I Slidebook Version 4.0 software (Optical Analysis, Nashua, New Hampshire, United States).

### s-Dectin-Fc constructs.

Two soluble fusion proteins consisting of the extracellular domain of murine dectin-1 fused with either the heavy chain of murine IgG1 (s-dectin-mFc) or a mutated Fc portion of human IgG1 (s-dectin-hFc) were constructed. For s-dectin-mFc, cDNA encoding the extracellular domain of dectin-1, consisting of amino acids 69 to 244 [25], was amplified from a PCR 3.1 plasmid encoding the full-length murine dectin-1 receptor using the primers GGGTACCGACGACACAATTCAGGG and GGATCCACGCGGAACCAGCAGTTCCTTCTCACAG. The cDNA encoding CH2-CH3 murine IgG1 regions were amplified using the primers CTGGTTCCGCGTGGATCCGTGCCCAGGGATTGTGGT and GAATTCTCATTTACCAGGAGAGTG from the pACCKP2 plasmid containing the TNF receptor extracellular domain linked to murine IgG1 heavy chain [43]. For the s-dectin-hFc construct, the pSecTag2 (Invitrogen) plasmid containing a mutated Fc portion of human IgG1 [38] was used. The products were combined at a 1:1 ratio, and PCR was performed using the 5′ dectin-1 primer and the 3′ IgG1 primer. The chimeric PCR product was isolated and purified via gel extraction and was subcloned into the TOPO-TA Vector (Invitrogen). Using M13 Forward and Reverse primers, the s-dectin-Fc DNAs were amplified via PCR and digested with KpnI and EcoRI and inserted in-frame into the multiple cloning site of pSecTag2 C mammalian expression vector (Invitrogen), containing the Igκ-leader sequence, facilitating protein secretion. To verify fusion protein expression, the pSecTag2 s-dectin-mFc or s-dectin-hFc constructs were transfected into HEK293T cells using Lipofectamine 2000 (Invitrogen). Western blotting of supernatants from transfected cells revealed a 120-kD product on non-reducing SDS-PAGE that reacted with either anti-human IgG1 or anti-murine IgG1. For analysis of the effects of s-dectin-hFc on cytokine and chemokine production, alveolar macrophages were co-cultured with *A. fumigatus* conidia at a ratio of 1:1 for 24 h in the presence or absence of s-dectin-hFc (10 μg/ml) in a 96-well plate at 37 °C, 5% CO_2_. Controls included alveolar macrophages cultured in medium alone. Thereafter, the contents of each well were collected and the supernatants analyzed for cytokines and chemokines by Bio-Plex (Bio-Rad, Hercules, California, United States).

### Analysis of *A. fumigatus*, beta-glucan exposure.

*A. fumigatus* RC were adhered for 2 h, 6 h, 10 h, or 24 h to sterile, round glass coverslips and incubated in the presence or absence of conditioned media containing s-dectin-mFc followed by Cy3-conjugated, goat anti-mouse IgG1. After being washed, the coverslips were mounted onto glass slides and Prolong mounting media (Molecular Probes) was applied. The coverslips were analyzed on a Zeiss Axioplan 2 upright fluorescent deconvolution microscope (Zeiss), and images were captured using 3I Slidebook Version 4.0 software.

### In vivo *A. fumigatus* challenge.

Mice were lightly anesthetized with isoflurane and held in a vertical, upright position. *A. fumigatus* conidia, 5 × 10^6^ in a volume of 50 μl, in the presence or absence of s-dectin-hFc (40 μg/ml) was administered to mice via the caudal oropharynx. At 24 h post-inoculation, mice were anesthetized with intraperitoneal pentobarbital, sacrificed by exsanguination, and a BAL was performed. The first ml of BAL fluid was collected, the supernatant clarified by centrifugation, and stored at −80 °C until use in Bio-Plex (Bio-Rad) assays. For total BAL-fluid cell determinations, the cell pellet from each individual sample was resuspended in 1 ml of tissue culture media and enumerated by a hemacytometer using trypan blue dye exclusion. To determine neutrophil counts in BAL fluid, 2.5 × 10^4^ cells from each lavage pellet was cytospun onto slides and stained with Diff-Quik (Fisher Scientific, Pittsburgh, Pennsylvania, United States). Thereafter, percentages of lymphocytes, macrophages, and neutrophils were determined in blinded fashion. To determine *A. fumigatus* lung burden, lungs were excised from non-lavaged untreated and s-dectin-hFc–treated mice and homogenized using a Polytron PT1200E tissue homogenizer (Kinematica, Newark, New Jersey, United States). Serial 1:10 dilutions were plated onto potato dextrose agar, and CFU/lung were determined after 24 h at 37 °C.

### Statistics.

Data were analyzed using StatView statistical software (Brainpower, Calabasas, California, United States). Comparisons between groups were made with analyses of variance and appropriate ad hoc testing. The two-tailed unpaired *t* test or the two-tailed nonparametric Mann-Whitney test was employed. Significance was accepted at *p* < 0.05.

## Supporting Information

Figure S1 Internalization of *A. fumigatus* by Alveolar Macrophages (142 KB PDF)

## Abbreviations

BAL: bronchoalveolar lavage
CSF: colony stimulating factor
GM: granulocyte monocyte
IL: interleukin
IPA: invasive pulmonary aspergillosis
LDH: lactate dehydrogenase
MIP: macrophage inflammatory protein
RC: resting conidia
SC: swollen conidia
s-dectin: soluble dectin
TNF-α: tumor necrosis factor α
TLR: toll-like receptor
