# Supplementary material for: The Beta-Glucan Receptor Dectin-1 Recognizes Specific Morphologies of Aspergillus fumigatus
Source: PLoS Pathog. 2005 Dec 9;1(4):e42. doi: 10.1371/journal.ppat.0010042 (PMC1311140; doi:10.1371/journal.ppat.0010042)
Supplement: Supplementary file 1 [file ppat.0010042.sg001.pdf]

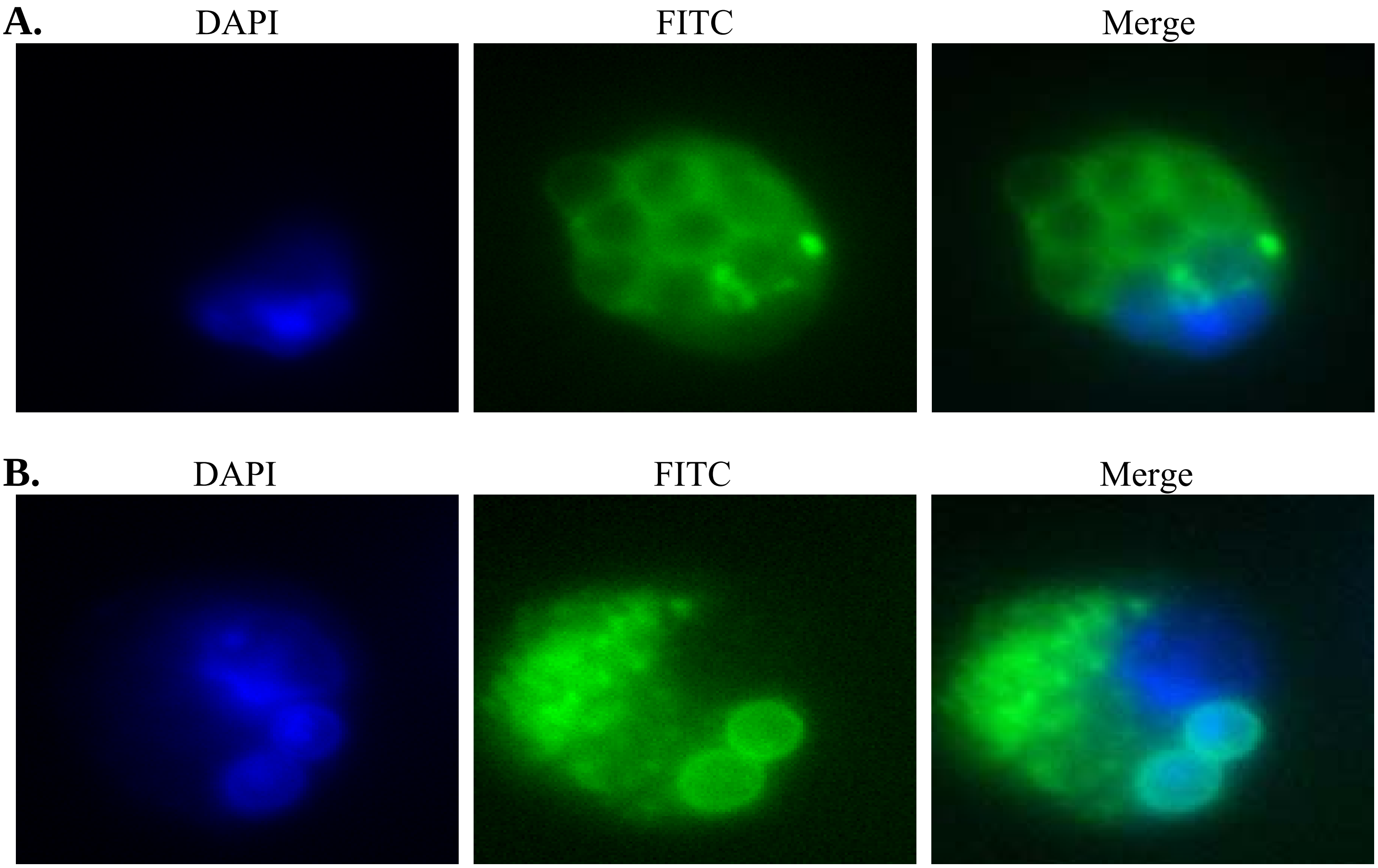

Supplemental Figure 1. Internalization of *A. fumigatus* by alveolar macrophages. Alveolar macrophages were adhered to glass slides and thereafter treated with dimethyl sulfoxide (DMSO – vehicle) or cytochalasin D (10 µM) for 1 h at 37°C. Fluoroscein isothiocyante(FITC)-conjugated *A. fumigatus* conidia were added at a macrophage to conidia ratio of 1:10 for 1 h at 37°C. Imaging was performed using a Zeiss Axioplan 2 upright fluorescent deconvolution microscope and images were captured using 3I Slidebook Version 4.0 software. Representative micrographs show vehicle **(A)** and cytochalasin D **(B)** treated alveolar macrophages. Left, middle and right images correspond to the nuclear stain DAPI, FITC and the merged image. Magnification is 630X oil emersion for all frames.
